# Optical Coherence Tomography Findings in Idiopathic Macular Holes

**DOI:** 10.1155/2011/928205

**Published:** 2011-07-18

**Authors:** Lynn L. Huang, David H. Levinson, Jonathan P. Levine, Umar Mian, Irena Tsui

**Affiliations:** Retina Division, Department of Ophthalmology, Montefiore Medical Center, Albert Einstein College of Medicine, The Bronx, NY 10467, USA

## Abstract

*Purpose*. To describe the characteristics of idiopathic macular holes (MH) on optical coherence tomography (OCT) and correlate OCT with clinical assessment. *Design*. Cross-sectional chart review and OCT assessment. *Participants*. Sixty-seven eyes with a clinically diagnosed idiopathic MH with available OCT data. *Methods*. A retrospective chart review and OCT assessment. *Results*. Based on OCT grading, 40 eyes had a full-thickness macular hole (FTMH) and 21 eyes had a lamellar macular hole (LMH). Clinical exam and OCT assessment agreed in 53 (87%) eyes when assessing the extent of MH. Six eyes (14.6%) in the FTMH group, and 3 eyes in the LMH group (14.3%) had persistent vitreomacular traction. Thirty-seven eyes (92.5%) in the FTMH group and 11 eyes (52.4%) in the LMH group had associated intraretinal cysts. Two eyes (5.0%) in the FTMH group and zero eyes in the LMH group had subretinal fluid. Intraretinal cysts were found to be more frequently associated with FTMH than with LMH (*P* < 0.001). *Conclusion*. This paper described OCT findings in a group of patients with clinically diagnosed MH. A high level of correlation between clinical assessment and OCT findings of LMH and FTMH was observed, and intraretinal cysts were often present in FTMH.

## 1. Introduction

For decades, macular holes (MH) have been classified by fundus biomicroscopy in four stages, as first described by Donald Gass in 1988 [1, 2]. The Gass classification is based on the fovea appearance, the estimated size of the hole, and whether or not the posterior vitreous is separated (Table 1). Confirmation of full-thickness macular holes (FTMH) can be performed with further clinical investigations such as Amsler grid assessment, the Watzke-Allen sign, or the laser aiming beam test [3]. 

Optical coherence tomography (OCT) has enhanced our understanding of MH by providing an objective and reproducible way of visualizing the macula. It confirmed the pathogenesis of idiopathic MH by introducing the concept of stage 0 macular hole, or vitreomacular traction (VMT) [4]. 

OCT provides confirmation of clinical findings, further anatomic characterization, means of educating patients, and improved staging of MH. In a study of 61 eyes with all stages of MH, OCT offered additional or different information in 92% of stage 1 MH [5]. Clinical assessment of late-staged MH is enhanced with OCT by enabling measurement of the diameter of the MH and visualizing the posterior hyaloid. 

The purpose of this study was to describe OCT findings of idiopathic MH in a series of consecutive patients seen at a single tertiary care practice.

## 2. Methods

A retrospective chart review was performed at the Henkind Eye Institute at the Montefiore Medical Center in Bronx, New York. Institutional Review Board approval was obtained for this study. All research was carried out in accordance with the Health Insurance Portability and Accountability Act of 1996. 

An initial search for the diagnosis of “macular hole” (International Classification of Diseases Ninth Revision 362.54) was done among 15,600 patient visits in the clinic database over a three-year time period. Charts were reviewed to confirm the clinical diagnosis of MH, and only idiopathic MH were included. Macular holes associated with trauma, retinal detachment, diabetic retinopathy, or myopia were excluded. Macular pseudoholes (MPH) associated with an epiretinal membrane by clinical diagnosis were also excluded. Patients with the initial clinical diagnosis of MH but no OCT were also excluded. Charts were reviewed for demographical (age, sex) and clinical information (visual acuity, clinical staging).

OCT was taken with time-domain OCT (Stratus OCT, Carl Zeiss Meditec, Inc., Dublin, CA, USA) using 6-line raster protocol or spectral-domain OCT (Cirrus OCT, Carl Zeiss Meditec, Inc., Dublin, CA, USA) using 5-line raster protocol. Assessment of OCT was based on both chart data and, when available, realtime imaging on the scanner. OCT review was done by two observers. OCT was evaluated for the extent of MH formation, for example, lamellar macular hole (LMH) versus FTMH. Additional OCT findings such as the presence of VMT seen as foveal vitreomacular adhesion, intraretinal cysts, and subretinal fluid (SRF) were evaluated. The relationship between OCT findings and the extent of MH and the agreement between clinical diagnosis and OCT diagnosis (LMH versus FTMH) were assessed using Chi-square tests. 

## 3. Results

An initial search by ICD-9 code revealed 133 patients with the diagnosis of MH. A total of 112 charts were available for review. Eighty eyes met inclusion criteria for idiopathic MH by clinical documentation. Among these eyes, 67 eyes had available OCT data at presentation. 

The mean age of patients was 71 years (range 35–97 years.) Forty-nine (69%) patients were female, accounting for a female-to-male ratio of 2.2 : 1. Best corrected visual acuity (BCVA) at presentation ranged from 20/25 to counting fingers (CF.) Forty-eight (60%) eyes had BCVA ≤ 20/200. Twenty eyes (25%) had BCVA ≥ 20/40. Twenty eyes were clinically diagnosed as LMH and 43 eyes as FTMH. The median BCVA was 20/70 in LMH group and 20/200 in FTMH group.

### 3.1. OCT Findings

Among 67 eyes with OCT data, 32 (48%) had TD-OCT, and 37 (55%) had SD-OCT. Four eyes were found to have epiretinal membrane with MPH and 2 eyes with resolved MH on OCT were excluded from the final analysis. Based on OCT grading, 40 eyes (66%) had FTMH and 21 eyes (35%) had LMH. Six (15%) of 40 eyes with FTMH by OCT evaluation had VMT versus 3/21 (14%) eyes with an LMH. Thirty-seven (93%) of 40 eyes with an FTMH by OCT evaluation had associated intraretinal cysts versus 11/21 (52%) eyes with a LMH. Two of 40 (5%) eyes with an FTMH by OCT evaluation had SRF versus zero eyes with an LMH. Intraretinal cysts were found to be more frequently associated with FTMH than in LMH (*P* < 0.001; Table 2).

### 3.2. Clinical Exam and OCT Agreement

Overall, clinical exam and OCT data agreed in 53 (87%) eyes when assessing the extent of IMH (LMH versus FTMH.) The rate of agreement was higher in the OCT-confirmed FTMH group (93%) than in the OCT-confirmed LMH group (76%) (*P* < 0.1; Table 2).

## 4. Discussion

**Figure 1:**
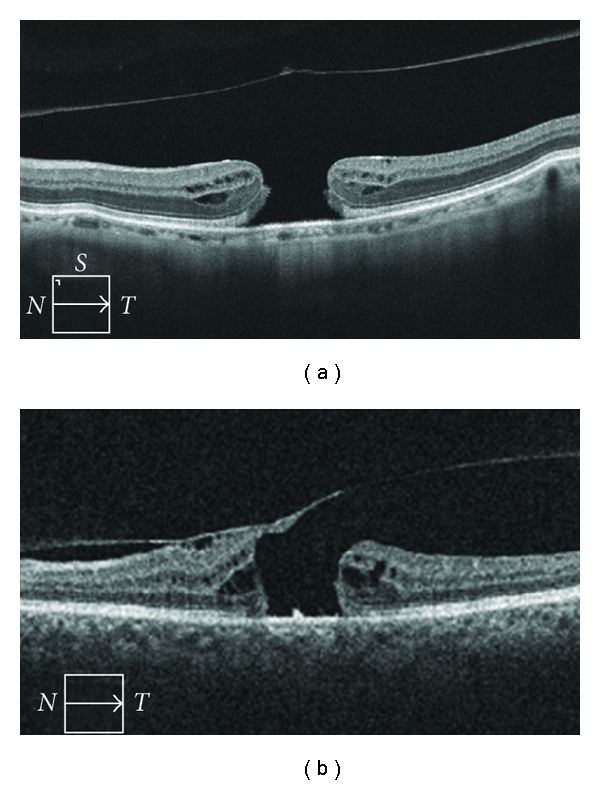
Optical coherence tomography of full-thickness macular holes (a) with separation of the posterior vitreous from the fovea and (b) with vitreomacular traction. Note the difference in foveal contour, as well as the presence of intraretinal cysts.

**Figure 2:**
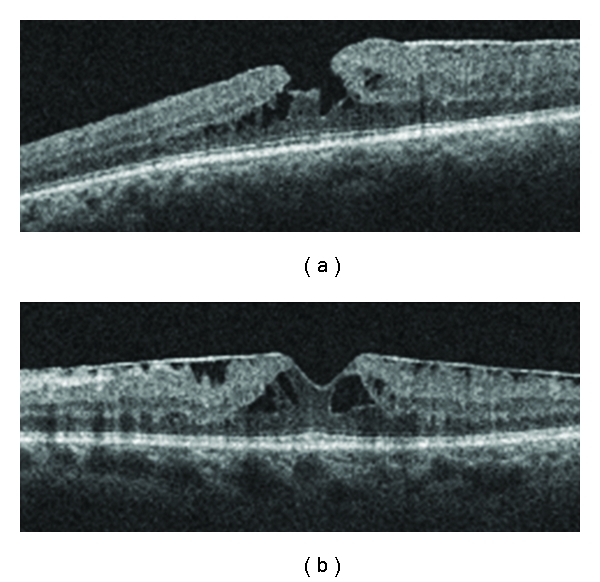
Optical coherence tomography of (a) a lamellar macular hole and (b) an epiretinal membrane with macular pseudohole. Note the difference in foveal contour, as well as intraretinal cysts.

**Figure 3:**
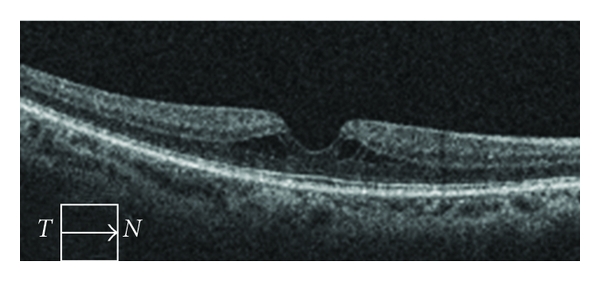
Optical coherence tomography of a lamellar macular hole with a smooth foveal contour. Notice intraretinal cysts.

This paper was a descriptive OCT study of idiopathic MH captured at a large tertiary care center. Besides distinguishing LMH and FTMH, OCT data provided additional information such as the presence of VMT, intraretinal cysts, and SRF. VMT occurred at about the same rate (14-15%) in both LMH and FTMH. An example of an FTMH with and without VMT is shown in Figure 1. Intraretinal cysts were found to be significantly more common in FTMH than in LMH. Figures 1–3 demonstrate intraretinal cysts; the presence of cysts on OCT in all stages of MH formation has been previously reported [6, 7]. SRF was uncommon in both LMH and FTMH among our patients. Fellow eyes should be imaged as well to look for subclinical VMT and intraretinal cysts, as these factors indicate increased risk of developing MH [6]. 

LMH were classified as a partial-thickness MH which showed an irregularity of the contour at the foveal center, and MPH as partial-thickness retinal defects associated with an epiretinal membrane and smooth configuration at the foveal center (Figure 2.) These two entities are generally considered different from each other [8, 9]. However, the distinction between MPH and LMH was not always clear. For example, one eye had a smooth contour at the foveal center without the presence of an epiretinal membrane (Figure 3). Furthermore, Michalewski et al. have reported a case of MPH that evolved into a lamellar IMH [10].

In the present study overall agreement between clinical examination and OCT was 87% and higher in the FTMH group than in the LMH group. However, clinicians were not blinded to the OCT results at time of clinical exam, thus the true rate of agreement may be lower, highlighting the utility of OCT in characterizing and staging MH. It would be interesting to assess the sensitivity and specificity of clinical examination for the diagnosis of MH, using OCT as the gold standard. Given its low incidence, however, this would require imaging a very large number of eyes. 

OCT has greatly advanced innovations in the treatment for MH by offering an objective means of assessing MH closure. For example, the necessity of internal limiting membrane (ILM) peeling, adjunctive ILM staining, and postoperative positioning are controversial concepts, which have recently been evaluated by OCT [11–13]. Clinical trials with microplasmin for the nonsurgical closure of FTMH also used OCT as a primary outcome [14].

A limitation of the present study is the retrospective data collection and use of two different OCT machines. Therefore, our OCT analysis was limited to subjective evaluation without quantitative comparisons. Although OCT provides anatomical information about the fovea, other imaging modalities, such as autofluorescence and fluorescein angiography, can provide functional information of the underlying retinal pigment epithelial cells [15, 16]. These additional tests can be considered for prospective studies on MH.

In summary this study described OCT findings in a group of patients with clinically diagnosed MH. A high level of correlation between clinical assessment and OCT findings of LMH and FTMH was observed; intraretinal cysts were often present in FTMH. Understanding of the etiology and management of MH has evolved with the use of OCT technology.

## Figures and Tables

**Table 1:** Modified Gass classification system of macular holes.

Stage	Description
Stage 1a	Yellow spot with loss of foveal depression, no vitreous separation

Stage 1b	Yellow ring with loss of foveal depression, no vitreous separation

Stage 2	Small full-thickness macular hole < 400 microns

Stage 3	Full-thickness macular hole > 400 microns, no vitreous separation

Stage 4	Full-thickness macular hole > 400 microns, complete vitreous separation

**Table 2:** Optical coherence tomography (OCT) summary (MH = macular hole; VMT = vitreomacular traction; IRC = intraretinal cysts; SRF = subretinal fluid).

	Additional OCT findings			Agreement with clinical exam (%)
OCT diagnosis	VMT (%)	IRC (%)	SRF (%)	
Total MH (*n* = 61)	9 (15)	48 (79)	2 (3)	53 (87)
Lamellar MH (*n* = 21)	3 (14)	11 (52)	0 (0)	16 (76)
Full-thickness MH (*n* = 40)	6 (15)	37 (93)	2 (5)	37 (93)
*P* value	>0.1	<.001	>0.1	<0.1
